# Interplay Between Mitochondrial Peroxiredoxins and ROS in Cancer Development and Progression

**DOI:** 10.3390/ijms20184407

**Published:** 2019-09-07

**Authors:** Tayaba Ismail, Youni Kim, Hongchan Lee, Dong-Seok Lee, Hyun-Shik Lee

**Affiliations:** KNU-Center for Nonlinear Dynamics, CMRI, School of Life Sciences, BK21 Plus KNU Creative BioResearch Group, College of Natural Sciences, Kyungpook National University, Daegu 41566, Korea (T.I.) (Y.K.) (H.L.) (D.-S.L.)

**Keywords:** mitochondria, peroxiredoxins, tumorigenesis, reactive oxygen species, ROS scavengers

## Abstract

Mitochondria are multifunctional cellular organelles that are major producers of reactive oxygen species (ROS) in eukaryotes; to maintain the redox balance, they are supplemented with different ROS scavengers, including mitochondrial peroxiredoxins (Prdxs). Mitochondrial Prdxs have physiological and pathological significance and are associated with the initiation and progression of various cancer types. In this review, we have focused on signaling involving ROS and mitochondrial Prdxs that is associated with cancer development and progression. An upregulated expression of Prdx3 and Prdx5 has been reported in different cancer types, such as breast, ovarian, endometrial, and lung cancers, as well as in Hodgkin’s lymphoma and hepatocellular carcinoma. The expression of Prdx3 and Prdx5 in different types of malignancies involves their association with different factors, such as transcription factors, micro RNAs, tumor suppressors, response elements, and oncogenic genes. The microenvironment of mitochondrial Prdxs plays an important role in cancer development, as cancerous cells are equipped with a high level of antioxidants to overcome excessive ROS production. However, an increased production of Prdx3 and Prdx5 is associated with the development of chemoresistance in certain types of cancers and it leads to further complications in cancer treatment. Understanding the interplay between mitochondrial Prdxs and ROS in carcinogenesis can be useful in the development of anticancer drugs with better proficiency and decreased resistance. However, more targeted studies are required for exploring the tumor microenvironment in association with mitochondrial Prdxs to improve the existing cancer therapies and drug development.

## 1. Introduction

Mitochondria, with their multifaceted roles, are recognized as indispensable organelles in eukaryotic cells and are considered as the energy currency of the cell because of adenosine triphosphate (ATP) production [1]. In addition to being energy sources, they are involved in heme synthesis, metabolism of amino acids, and the regulation of the redox state of cells [2,3]. These multiple functions of mitochondria make them prerequisites for cellular life of eukaryotes [4]. Apart from these functions that are related to cell survival, a large body of evidence has exhibited that this subcellular organelle also has crucial functions in the cell death program [5,6]. Mitochondria play significant roles in apoptosis by regulating the release of pro-apoptotic factors [7]. In addition, they are also critical participants in necrosis and autophagy [8,9].

Mitochondria continuously communicate with other cells by signal transduction to carry out this diverse array of functions [10,11]. The generation of reactive oxygen species (ROS) is one of the way of mitochondrial signal transduction and these generated (ROS) function as secondary messengers [12,13] and play a significant role in cellular signal transduction [14]. ROS are basically short-lived species having unpaired electrons [15,16], and they are endogenously generated as a byproduct during mitochondrial energy production as well as produced as a consequence of fatty acid β-oxidation and exposure to radiation, light, metals, and redox drugs [17]. Mitochondria being the largest contributors of ROS in mammalian cells convert approximately 1% of their consumed oxygen to superoxide anion (O^2−)^ [18], and they have up to ten sites with ROS generation ability [19]. ROS serve as secondary messengers by interacting with a variety of molecules; they are important for many biologically significant processes, such as adaptive immunity, cell differentiation, and oxygen cell sensing [13,15,16,20,21]. Mitochondrial ROS, in addition to their cellular signaling properties, also have cell damaging roles [22]. 

**Figure 1:**
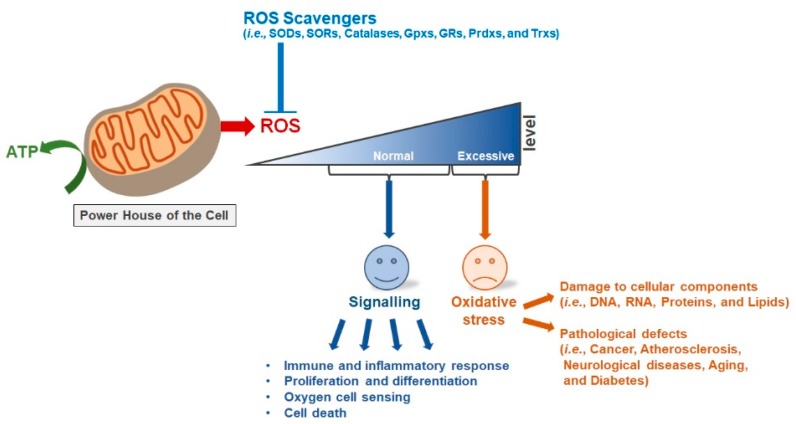
Mitochondrial reactive oxygen species (ROS) scavengers and cancer development. Eukaryotic cells depend upon the mitochondria for energy production, and reactive oxygen species (ROS) are produced as byproducts during adenosine triphosphate (ATP) generation by the mitochondria. The amount of mitochondrial ROS is balanced by ROS scavengers in the mitochondria of normal eukaryotic cells and these ROS regulate different cellular processes such as cell proliferation and differentiation by acting as signaling molecules. The loss of this specific balance between ROS and ROS scavengers leads to an outburst growth of cells, resulting in cancer initiation and development.

Therefore, ROS homeostasis is essential for steady state functions of cells [23,24]. The accumulation of ROS resulting from any imbalance in ROS production and metabolism induces oxidative stress [25]. In other words, oxidative stress is the result of imbalance between two opposite and opposing forces, i.e., ROS production and antioxidation, and it can lead to pathological defects in living organisms, such as cancer, atherosclerosis, neurological diseases, aging, and diabetes, as well as can harm the cellular components, such as DNA, RNA, lipids, and proteins [26,27]. To protect cells from the oxidative stress, there are many enzymatic and non-enzymatic defense systems in mitochondria [28,29]. The non-enzymatic defense system includes flavonoids, vitamins (A, C, and E), and glutathione [28]. Superoxide dismutase (SOD), superoxide reductase, catalase, glutathione peroxidase, glutathione reductase, peroxiredoxins (Prdxs), and thioredoxins (Trx) are important enzymatic antioxidants that are involved in the regulation of mitochondrial ROS [30,31]. ROS production in mitochondria and their subsequent fate involve interactions between various molecules in normal and pathological conditions [32] (Figure 1).

The aim of the present review is to focus on the signaling for ROS regulation involving mitochondrial Prdxs in normal as well as disease conditions. Mitochondrial Prdxs are recently discovered antioxidants presenting a variety of roles in eukaryotic organisms. Recently, mitochondrial Prdxs have gathered much interest due to their diversified functions and interactions in living organisms. In the present review, we describe the factors that are associated with mitochondrial Prdxs in ROS scavenging, as well as its signaling pattern in normal and pathological cellular conditions.

## 2. Mitochondrial Prdxs

Prdxs denote the family of proteins having the ability to efficiently scavenge peroxides in the form of hydrogen peroxide, alkyl hydro peroxide, and peroxynitrite [33]. Prdxs are classified into six subfamilies, according to PeroxiRedoxin classification index (PREX) and, in mammals, there are six Prdxs, among which Prdx 1–4 belongs to Prdx1 subfamily, Prdx5 is a member of Prdx5 subfamily, and Prdx6 represent Prdx6 subfamily [34,35]. Prdxs are also classified as 1-Cys containing only Prdx6, while typical 2-Cys comprised of Prdx1–4 and Prdx5 belongs to atypical 2-Cys on the basis of their cysteine residues and their catalytic mechanism [36]. The catalytic activity of Prdxs is highly dependent upon the conserved peroxidatic cysteine (Cp) in the amino terminal region of the protein. In addition to this conserved Cp, there is an additional conserved cysteine residue that is present in the carboxyl terminal portion of the five out of six mammalian Prdxs, termed as resolving cysteine (Cr). [37]. Prdxs belonging to typical 2-Cys class are typical homodimers containing two identical active sites that brings peroxidatic and resolving cysteines (Cp and Cr) into juxtaposition. Peroxidatic cysteine of typical 2-Cys is oxidized by peroxide, resulting in the formation of sulfenic acid (C-SOH), which in turn condenses with Cr of opposite subunit, and results in the formation of intermolecular disulfide bond. The oxidized Prdxs are reduced by appropriate electron donor to complete the catalytic cycle. The oxidized Cp of atypical 2-Cys condenses with Cr within same polypeptide to form intramolecular disulfide bond and in turn reduced by the Trx2. In contrast to typical and atypical 2-Cys Prdxs, 1-Cys Prdxs has only one cysteine residue and depends on glutathione (GSH) to complete the catalytic peroxidatic cycle [38]. Members of typical 2-Cys class have the ability to form decamers and do-decamers in their reduced or hyperoxidized state. This feature enables them to function as chaperons, enzyme activators, and redox sensors, in addition to their antioxidative abilities [39]. While, atypical 2-Cys Prdxs can undergo protein-protein interaction in combination with their antioxidative properties [40]. In contrast, 1-Cys Prdxs cannot form decamers, and mainly functions as antioxidant rather than molecular chaperons, although their antioxidation catalytic mechanism is similar to typical 2-Cys [41]. 

Mammalian Prdxs also differ in their cellular location and are distributed in cytosol, mitochondria, endoplasmic reticulum, peroxisomes, and nuclei [42]. Based on subcellular distribution, Prdx3 and 5 are categorized as mitochondrial Prdxs [43]. Studies on mitochondrial Prdxs were initiated in 1989 with the cloning of the *mer5* gene [44]. This gene is expressed in murine erythroleukemia [45]. Prdx3 was thought to play a significant role in erythrocyte differentiation [46]. This gene was further investigated to have considerable sequence identity with bacterial alkyl hydroperoxide reductase (AhpC) [47]. Meanwhile, substrates of mitochondrial ATP-dependent protease, later identified as Lon protease, were investigated, leading to the discovery of a 22-kDa substrate named as SP-22 [48]. This SP-22 was found to be a highly abundant protein in the mitochondrial matrix, sharing a sequence homology of 90% with Mer5 and having considerable identity with AhpC. It is now recognized as “Prdx3”. Prdx3 was also termed as an antioxidant protein 1 (AoP1) [48]. Simultaneously, when the *mer5* gene was cloned, a novel gene, *PMP20*, was identified in *Candida boidinii,* encoding a peroxisomal membrane-associated protein [49]. The homologue of *PMP20* was discovered 10 years later in humans with thiol-specific antioxidant properties [50]. In the same year, a novel gene, named “AEB166”, having a sequence identity of 65% with bacterial Prdx, was identified [51]. This gene was later termed as “Prdx5” [52]. 

## 3. Characteristics of mitochondrial Prdxs

**Figure 2:**
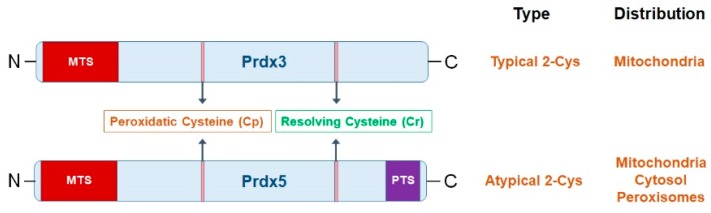
Structural comparison of mitochondrial peroxiredoxins (Prdxs). Peroxiredoxin 3 (Prdx3) and Peroxiredoxin 5 (Prdx5) contain a mitochondrial targeting sequence (MTS) at their amino terminal (N-terminal) and this enables them to reside in the mitochondria. Prdx5 in addition to MTS also contains a peroxisomal targeting sequence (PTS) at its carboxyl terminal (C-terminal) that is responsible for the entry of Prdx5 in the peroxisomes. Both mitochondrial Prdxs comprise peroxidatic cysteine (Cp) and resolving cysteine (Cr) which play an essential role in their catalytic mechanisms. In Prdx3, Cp is located at residue 47, whereas, in Prdx5, it is located at residue 48 [35].

Prdx3 is located on chromosome 10 (q25–q26) and it is transcribed from seven exons in humans. It is a 256-amino acid containing protein with a 61-amino acid long mitochondrial targeting sequence at the N-terminal. The cleavage of this mitochondrial sequence results in a 21.5-kDa protein that resides in the mitochondrial matrix [37]. Peroxidatic cysteine (Cp) is located at the residue 47 and it is a highly conserved region of the protein. Prdx3 belongs to “typical 2-Cys” class of Prdxs, i.e., it is a homodimer organized in a head to tail manner and during catalysis, utilizes Cp and resolving cysteine (Cr) residues on opposite subunits [43] (Figure 2). 

In contrast to Prdx3, human Prdx5 is located on chromosome 11 (q13) and it is transcribed from six exons. It contains a 52-amino acid-long mitochondrial targeting leader sequence at its N-terminal and it also has a C-terminal SQL sequence that is specific for peroxisomes [52] (Figure 2). This peroxisomal targeting sequence enables this protein to localize in both peroxisomes and mitochondria [53]. The cleavage of mitochondrial targeting sequence yields a 17-kDa protein having Cp at residue 48 of the mature protein. The active site of Prdx5 has several sequences in common with other Prdx family members [54]. The human Prdx5 has a divergent sequence sharing a sequence homology of 28–30% with Prdx1, Prdx2, and Prdx3. Prdx5 is the only mammalian Prdx that belongs to “atypical 2-Cys” subfamily of Prdxs [55](Figure 2). 

## 4. ROS and Mitochondrial Prdxs

ROS are extraordinarily active oxygen species involving superoxide anion (O^2−^), hydroxyl radical (OH**^·^**), and hydrogen peroxide (H_2_O_2_) [56]. They are generated by the partial reduction of oxygen, and comprise radical and non-radical oxygen species [56]. Endogenous sources of ROS include mitochondrial electron transport linked phosphorylation, P450 metabolism, peroxisome metabolism, and activation of inflammatory cells [13]. Mitochondrial ROS are produced in mitochondrial electron transport chain during ATP generation [13,15]. 

Previously, ROS were considered to be damaging to cells because of their association with oxidative stress, which leads to the induction of pathological responses [14]. However, in the past few decades, ROS have been recognized as signaling molecules that regulate different processes of biological and physiological importance [57]. H_2_O_2_ is the ROS that directly serves as a secondary messenger and is majorly produced by the mitochondria [58,59]. Redox signal transduction involves the oxidation of Cys residues in the proteins that is mediated by H_2_O_2_ [58]. At physiological pH, Cys residues exist in the form of thiolate anions (Cys-S^−^) and are more receptive to oxidation as compared to protonated Cys thiols (Cys-SH) [60]. During the process of redox signaling, Cys-S^−^ is oxidized to its sulfenic form (Cys-SOH) by H_2_O_2_, which leads to allosteric alterations in the protein, and ultimately changes its function [61,62]. Thioredoxin and glutaredoxins can reduce back this sulfenic form of Cys to thiolate anion and bring back the protein to its original form [63]. Thus, the oxidation of Cys residues in proteins involves a reversible signal transduction mechanism that mostly occurs at nanomolar concentrations of H_2_O_2_, whereas at higher H_2_O_2_ concentrations, thiolate anions are further oxidized to sulfinic (SO_2_H) and sulfonic (SO_3_H) species [62,63]. Sulfiredoxin (Srx) can slowly reduce back the SO_2_H form of Prdxs to its native enzyme [64]. In contrast to Cys-SOH and SO_2_H species, the oxidation to sulfonic species can be irreversible, which results in damage to the protein (oxidative stress) [61,65]. Mitochondria and cells are equipped with professional enzymes to prevent the accumulation of H_2_O_2_, particularly Prdxs and glutaredoxins [66]. 

Mitochondrial Prdxs play a significant role in H_2_O_2_ scavanging and to maintain a tight balance of H_2_O_2_ [67]. Mitochondrial Prdxs that belong to the typical and atypical 2-Cys subfamilies contain a redox sensitive cysteine at the active site that is oxidized by H_2_O_2_ and then reduced by thioredoxins to complete the catalytic cycle [68]. The oxidized thioredoxins are reduced by thioredoxin reductase at expense of NADPH [69]. The discriminative characteristic of Prdxs is their ability to undergo reversible hyperoxidation by a second molecule of H_2_O_2_, which results in the formation of sulfinic acid that consequently leads to the transient inactivation of the protein [70]. The reduction of the hyperoxidized form of Prdxs to SO^−^ is catalyzed by sulfredoxins through an ATP-dependent mechanism [70]. 

## 5. ROS and Mitochondrial Antioxidants in Oncogenic Signaling

**Figure 3:**
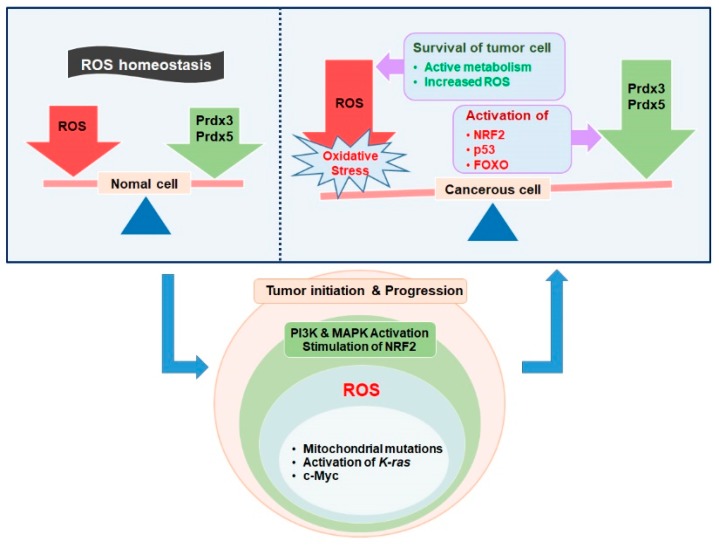
Role of antioxidants in cancerous cells. Mutations in mitochondria and mitochondrial dysfunctions lead to the production of increased amount of ROS by the activation of c-Myc and K-ras factors. The increased production of ROS leads to an imbalance between ROS and its scavengers. This imbalance activates a number of signaling pathways such as PI3K and mitogen-activated protein kinase (MAPK), resulting in tumor initiation and progression. The increased amount of ROS in cancerous cells requires an upregulated expression of antioxidants to prevent cell death. Thus, the activation of certain transcription factors (NRF2 and FOXO) and tumor suppressors (p53) induces the upregulation of mitochondrial Prdxs to compensate the increased production of ROS. Thus, mitochondrial Prdxs along with other scavengers act to protect the cancerous cells from ROS-mediated cell death.

Many vital biological processes, such as cellular signaling, metabolism, and epigenetics, are significantly regulated by ROS and, consequently, these biologically active oxygen species are involved in disease initiation and progression [15,30]. Mitochondria being one of the major source of ROS are associated with the roles of ROS in the living organisms and are significantly involved in the regulation of ROS to maintain a steady state cellular environment and prevent cells from oxidative damage [71]. ROS, particularly H_2_O_2_, is a byproduct of mitochondrial energy generation and is regulated by mitochondrial antioxidants [72]. Altered cellular metabolism is associated with the production of cancerous cells [73]. Cancerous cells are characterized by a high rate of proliferation and active metabolism [73]. These cancerous cells require higher energy levels for aberrant cellular proliferation and to maintain the high growth rate, these cells hijack the machinery of normal cells [74,75,76]. Growth-related pathways are constitutively activated in cancer cells and, consequently, these cells take up excess amount of nutrients, endure stress, and multiply rapidly [77,78]. This results in hyper metabolism, which leads to an excessive generation of ROS by the mitochondria, endoplasmic reticulum, and the action of NADPH oxidases [79]. Mitochondrial ROS are required for the proliferation of cancerous cells driven by K-ras oncogenes [80,81]. Mutations in the mitochondria that result in dysfunctions of TCA cycle/electron transport chain produce excess amount of ROS to trigger tumor associated signaling pathways, such as PI3K and MAP kinase signaling pathways [82,83,84]. In addition to these signaling pathways, ROS also target the transcription factor NF-kB (earliest discovered transcription factor responsive to ROS) that is associated with the survival of tumor cells [85,86] (Figure 3). 

Studies have shown that the association of ROS with cancer progression and suppression is dependent on their intracellular levels [87]. At low levels, ROS augment cancer proliferation by working as signal transducers or by prompting the genomic DNA alteration or damage to DNA [88]. For example, ROS can stimulate cyclin D1 expression [89,90], lead to the promotion of extracellular-signal-related kinase (ERK) Jun N–terminal kinase (JNK) phosphorylation [91,92], and trigger mitogen-activated protein kinase (MAPK) activation [93], all of which are associated with carcinogenesis and the survival of cancerous cells [94]. In addition to the functions of ROS in the pathways associated with cell proliferation, ROS have been recognized to inversely debilitate tumor suppressors, such as protein tyrosine phosphatases (PTPs) and phosphatase and tensin homolog (PTEN), because of the presence of redox-sensitive cysteine in their catalytic sites [95]. PTPs also function to regulate signaling pathways by inducing antioxidant enzyme expression and decreasing ROS levels [96,97]. Moreover, ROS act as regulators of normal stem cell renewal and differentiation. Cancerous stem cells (CSCs) exhibit similar characteristics; however, there is limited knowledge regarding their association with maintenance of cellular redox balance [98]. Recent studies have exhibited low ROS levels in liver and breast cancer stem cells, consequently resulting in the upregulation of ROS-scavenging signaling proteins expression [99]. If growth of CSCs is indispensable for tumor induction, maintenance of low ROS levels might be a prerequisite for pre-neoplastic foci tolerance [98]. Chemo and radiotherapies stimulate ROS production and they are useful for demolishing most cancerous cells [98,99]. Certain patients are unable to cure by the chemo and radio therapies because of increased endurance of CSCs to conditions of high ROS levels by augmented antioxidant levels [100]. Some adverse effects of anticancer drugs are debatably mediated by ROS; thus, CSCs may be appropriately released and vigorously chosen by the factors, depending on increased ROS levels [101]. Moreover, further oxidative stress that is mediated by these factors may result in additional mutations and DNA damage, ultimately leading to enlargement of drug-resistant cancer cells [98,101]. Previously, it was assumed that increased ROS generation in cancerous cells leads to genomic instability, and consequently promotes tumor formation [102]. However, genomic instability is associated with a loss of p53 and other processes that are related to aneuploidy [103,104]. ROS-dependent oncogenesis driven by Myc oncogenes in cancer cells exhibits no chromosomal instability [105,106].

ROS at high levels mediate cell death and cause adverse damage to the cells [107]. Therefore, cancerous cells must control increased ROS levels, specifically during the initial stages of cellular proliferation [108]. In cancerous cells with elevated ROS levels, redox balance is maintained by equally high amounts of antioxidants [109]. These antioxidants control the ROS levels and prevent the cells from cell death mediated by oxidative stress [110]. Recently, it has been discovered that conditions that facilitate oxidative stress also induce particular pressure on pre-neoplastic cells to trigger effective antioxidant mechanisms [111]. This characteristic is relevant, particularly during metastasis, which is characterized by the spread of cancer cells to distant organs [112]. Thus, cancer cells have extraordinary antioxidant capabilities to circumvent elevated ROS levels and they are accustomed to the diverse biological functions of cells [113]. The antioxidant capabilities of cancer cells are quite high when compared with those of normal cells [21,73,114]. The elevated antioxidant level in cancerous cells is achieved by the activation of transcription factors, such as nuclear factor erythroid 2 related factor 2 (NRF2) [115,116], that interacts with kelch-like ECH-associated proteins (KEAP1) and targets proteosomal degradation [117]. ROS sensitive Cys residues of KEAP1 are oxidized by elevated ROS levels, leading to KEAP1 dissociation from NRF2 [118]. The translocation of dissociated NRF2 to nucleus and its heterodimerization occurs with a small MAF protein [119]. Subsequently, it binds with antioxidant-responsive elements (AREs) of various antioxidative genes [119,120]. Although NRF2 protects the cells from cancer causing agents, it also stimulates tumor formation and cancer development by safeguarding cancer cells from ROS and the resulting DNA damage [108,115,121]. In some tumor cells, KEAP1 mutations lead to the constitutive NRF2 activation [122]. The loss of NRF2 induces oxidative stress in cancerous cells, and ultimately inhibits tumor formation [123]. The loss of NRF2 inhibits multiple antioxidant signaling pathways, resulting in damage to the cancer cells [124] (Figure 3). 

Elevated ROS are associated with tumor formation and progression. The tumor suppressive genes can serve as antioxidants for scavenging ROS and to maintain them at the levels that do not promote tumor formation [125]. Tumor suppressor p53 regulates the expression of many antioxidant genes [126,127]. Moreover, mice that are deficient in p53 have demonstrated a reduction in tumor formation by dietary supplement NAC, which suggests that, in certain cancers, the primary suppressive function of p53 is to reduce ROS levels [128]. However, it is also suggested that a major tumor suppressive function of p53 is to regulate the antioxidative and metabolic genes [129]. One mechanism by which p53 regulates the metabolism to control antioxidant function is the induction of p53 target TIGAR expression [130]. TIGAR, also known as 2, 6-fructose bisphosphatase, reduces the levels of fructose 2, 6-bisphosphate (a positive regulator of phosphofructokinase 1) [131]. Consequently, it leads to a reduction in glycolytic flux by shunting the glycolytic carbons to the pentose phosphate pathway to generate NADPH and the generated NADPH is required for the maintenance of the antioxidant system [132]. Other tumor suppressor genes such as FOXO transcription factors, function by activating many antioxidant genes, such as Prdx3 and Prdx5 [133,134]. 

ROS also regulate mitogenic signals to promote cancer cell proliferation and also play a role in adapting to metabolic stress condition when a highly proliferative tumor tissue outstrips its blood supply [135,136]. Hypoxia-inducible factors (HIFs) are stabilized in the resulting hypoxic tissue [137,138]. Prolyl hydroxylases (PHDs) hydroxylate proline residues of HIF1α that are recognized by E3 ubiquitin ligase von Hippel-Landau (pVHL) protein [139]. This causes HIF1α to proteasome degradation [140]. Non-hydroxylated HIF1α is not recognized by pVHL and translocates to the nucleus [139,141]. In the nucleus, it dimerizes with HIF1β and then regulates the metabolic adaption to hypoxia and the expression of pro-oncogenes, such as vascular endothelial growth factor (VEGF) [142]. HIFs that are induced by ROS promote tumorigenesis in certain cancer cells [143]. Moreover, sirtuin proteins (SIRT3) upregulate the antioxidant system to prevent HIF activation [144,145].

In summary, two counter signaling mechanisms operate in cancerous cells to maintain tumor proliferation and tumor survival. One type of signaling is associated with excessive ROS generation that results from the highly active metabolism of cancerous cells and the other is to counter balance this excessive ROS and to prevent the cancerous cells from oxidative damage that involves the activation of the antioxidant defense system. Furthermore, increased ROS levels by endogenous sources are dangerous for cancerous cells, as well as for cancer development [109]. Thus, these antioxidant protective mechanisms can be targeted to kill cancer cells along with CSCs while protecting the normal cells and considered to be a better approach for cancer treatment and therapy [146,147].

## 6. Prdx3 and Carcinogenesis 

**Figure 4:**
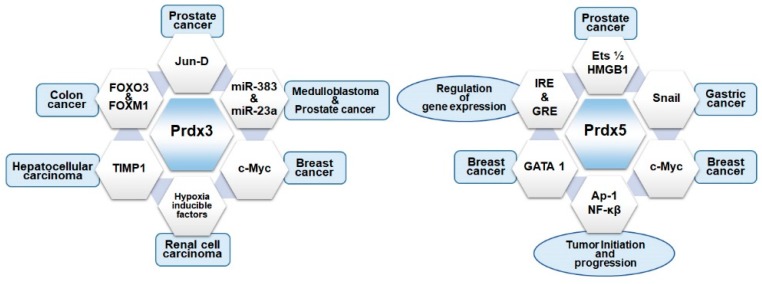
Microenvironment of mitochondrial Prdxs in cancer. The involvement of mitochondrial Prdxs in cancer depends on their interaction with different factors including transcription factors, different response elements, micro RNAs, and cancer-related genes. The microenvironment of Prdx3 involves its regulation by various transcription factors such as FOXO family transcription factors, Jun-D, and TIMP1 in colon, prostate, and hepatocellular carcinomas. These factors are associated with the upregulation of Prdx3 in the cancerous cells for the abovementioned cancer types. Prdx3 interacts with miR-383 and miR-23b in medulloblastoma and prostate cancer, further regulated by c-Myc in breast cancer and hypoxia-inducible factors in renal cell carcinoma. Similarly, the expression of Prdx5 in different malignancies depends on its regulation by a variety of interacting partners such as c-Myc and GATA1 in breast cancer, Snail in gastric cancer, and Ets1/2 and HMGB1 in prostate cancer. Additionally, Prdx5 interacts with transcription factors AP-1 and NF-κB, insulin and glucocorticoid response elements for the initiation of tumorigenesis and tumor progression.

As mentioned in Section 5, the cancer cells are endowed with extraordinarily high antioxidant capabilities; thus, it is not surprising that cancer cells contain upregulated levels of mitochondrial Prdxs [148]. Prdx3 is highly sensitive to oxidative stress, and is regulated by sirtuin1, a class III histone deacetylase (SIRT1). SIRT1 regulates the expression of Prdx3 by enhancing the formation of PGC-1α/FoxO3a transcriptional complex [149]. The signaling and regulation of Prdx3 varies, depending on the type of cancer and its interacting partners [150] (Figure 4). The debatable issue is whether these Prdxs function as cancer promoters or suppressors. Multiple lines of studies have shown that Prdx3 is upregulated in different types of cancerous cells [151,152,153]. The overexpression of Prdx3 is observed in colon cancer stem cells (CSCs) having elevated mitochondrial functions [150]. A positive correlation for upregulated expression of Prdx3 and CD133 is reported along with FOXM1 regulating the expression of Prdx3 and CD133 by binding to the promoter regions of both Prdx3 and CD133. Prdx3 knock out mice have shown a reduction in tumor volume and metastasis giving a clue for association of Prdx3 with FOXM1 associated pathways for cancer development [150]. FOXM1 is associated with cancer cell proliferation and the FOXM1/Prdx3 pathway plays a role in the survival of cells, so they can be specifically targeted to develop the efficient drugs. This study is significant in that both in vitro and in vivo results showed that Prdx3 is regulating the survival and metastasis of cancer stem cells through mitochondrial stabilization and the depletion of Prdx3 can lead to reduce tumor size and promote cell death by mitochondrial dysfunctions. The upregulated expression of Prdx3 is also observed in medulloblastoma (MB), along with the decreased expression of miR-383 [154]. RNA and protein levels of Prdx3 are both considerably reduced upon miR-383 restoration. This study provides clear evidence for the interaction of miR-383 with Prdx3 through miR-383 seed region in 3′ UTR of Prdx3 and reported the inverse relationship between miR-383 and Prdx3 in MB cells. However, this inverse relationship was not observed in MB samples [154,155]. This difference between Prdx3 and miR-383 relationship in MB cells and MB samples implies the need for in-vivo studies to further enhance the understanding of Prdx3 roles in cancer cell survival and metastasis. In addition to miR-383, the association of Prdx3 with miR-23b is also observed in prostate cancer (PCa). The PCa-cell line studies showed that Prdx3 is regulated by miR-23b in normal as well as in hypoxic conditions. miR-23b itself regulated by c-Myc in-turn regulates the expression of Prdx3 at both RNA and protein levels [156]. 

The association of Prdx3 with JunD is also observed in PCa, which indicates that JunD regulates cellular proliferation in PCa by regulating the expression of its associated genes, including Prdx3 with Myc family genes, as crucial downstream regulators [157]. These studies show that the involvement of Prdx3 in cancer is for more complicated as in PCa, the Prdx3 expression is regulated by miR-23b as well as by JunD. It is clear that Prdx3 is regulated by multiple factors and there is a dire need to analyze the co-relational regulation of Prdx3 by transcriptional factors and micro RNAs to identify the associated changes on the tumor microenvironment. 

The involvement of Prdx3 with hypoxia is also established and it is interesting to note that the overexpression of Prdx3 suppressed the hypoxia mediated apoptosis of thymoma cells in vitro, but with regard to the role of von Hippel-Laundau protein (pVHL) in clear-cell renal cell carcinoma, the protein level of Prdx3 is downregulated and HIF-1α stabilization is induced by pVHL deficiency in conditions of normoxia or hypoxia; this leads to decrease in Prdx3 expression and is associated with cellular proliferation of clear cell renal cell carcinoma (CCRCC) [158]. Taken together, these studies demonstrate the important role of Prdx3 in hypoxia, which is associated with cancer development and the response to cancer therapies.

The upregulated expression of Prdx3 is associated with an enhanced expression of ATP synthase and increased ATP production in hepatocellular carcinoma, and it plays a role in tumor growth and progression. However, at the same time, the downregulation of Prdx3 results in enhanced invasive properties of HepG2 cells through the downregulation of TIMP metallopeptidase inhibitor 1 (TIMP-1) and causes increased extracellular matrix (ECM) degradation [159,160,161]. This implies that the differential response of Prdx3 expression is regulated by diverse signaling pathways that are associated with cancer progression. 

Breast cancer is one of the most common cancer among females with many subtypes and it is associated with a high mortality rate if not diagnosed at early stages [162]. Proteomic analysis of invasive ductal carcinoma of the breast with luminal B human epidermal growth factor receptor 2-positive (HER2-positive LB) and HER2-enriched (HE) subtypes have shown that Prdx3 with an upregulated expression of luminal B HER2 can serve as a promising bio-signature for LB subtype and it can also serve as potential biomarker for the diagnosis of early- and late-stage disease [163]. Enhanced expression of Prdx3 is also observed in MCF-7 cells [162,164,165]. In cervical cancer, single nucleotide polymorphism of Prdx3 leads to significant increased risk of cervical cancer and progression [166]. In addition, gene expression analysis has revealed the increase in the expression of Prdx3 in cervical cancer [166]. Endometrial cancer is among the common cancers in females that affect the genital tract. The expression of Prdx3 is upregulated in the endometrium of patients that suffer from this type of cancer as compared to that in normal endometrium [167]. A high expression of Prdx3 is associated with endometrial cancer and it has the potential to serve as a prognostic marker for endometrial cancer; thus, it can be targeted for the development of better therapeutic strategies [151,168]. The upregulated expression of Prdx3 is also observed in malignant mesothelioma (MM) cells and an overexpression of Prdx3 in MM cells maintains a redox set point that enables these cells to survive in conditions with elevated levels of mitochondrial ROS [169]. Any disturbance in this Prdx3 regulated redox set point impairs cellular proliferation by affecting the cell cycle dynamics operating between energy metabolism and mitochondrial network [170]. Taken together, the upregulated expression of Prdx3 is observed in different cancerous cell lines but the mechanistic details are lacking in how the upregulated expression affects the environment in cancerous cells. 

Our survey of studies analyzing the expression of Prdx3 in human cancers clearly shows that Prdx3 is upregulated in many cancers and it is associated with cell proliferation. Hence, Prdx3 can be considered as pro-cell survival, either in healthy or diseased cells, and it can be targeted for cancer treatment. The cancerous cells have significantly high levels of ROS, irrespective of the upregulated expression of Prdxs as compared to the normal cell and these cancerous cells cannot respond to the increased ROS levels, like normal cells having many compensatory mechanisms to respond to ROS. Therefore, Prdxs inhibition can lead to a further increase in ROS and consequently can promote cell death of cancerous cells but not the normal cells having protective mechanisms [171]. However, there is evidence that downregulation of Prdx3 led to enhancing the tumor malignancies and invasiveness in certain cancers, so the targeting of Prdx3 for effective drug development requires further research and it will be beneficial to target the signaling pathway instead of a single factor. 

## 7. Prdx5 and Carcinogenesis

Mitochondrial oxidants are produced in significantly large amounts in cancerous cells due to oncogenic transformation and metabolic reorganization [172]. Like Prdx3, the up- and downregulation of Prdx5 is also observed in many types of cancers (Figure 4). The expression of Prdx5 is regulated by different transcription factors, including AP-1, nuclear factor-κB (NF-κB), antioxidant response element (ARE), insulin response element (IRE), glucocorticoid response element (GRE), and c-Myc; further, c-Myc might directly regulate Prdx5 expression by interacting with putative responsive elements in the 5’-flanking region of the gene [54,173]. Despite these other transcription factors, such as nuclear respiratory factor 1 (NRF1) and nuclear respiratory factor 2 (NRF2; GABPA), which are associated with the mammalian cells, response to oxidative stress and mitochondrial biogenesis are also capable of indirectly regulating Prdx5 expression [53,174]. c-Myc not only directly regulates Prdx5 transcription, but also participates in the establishment of ROS homeostasis by selectively inducing the transcription of specific Prdxs when the function of one of the Prdxs is compromised [175]. In microenvironmental stress conditions, such as hypoxia, E-twenty-six transcription factor 1 and 2 (Ets1/2), and high-mobility-group protein B1 (HMGB1), mediate the upregulation Prdx5 in cancer cells, particularly in human prostate and epidermoid cancer cells exposed to H_2_O_2_ or hypoxia [176]. The interaction of Prdx5 with a variety of regulators complicates its functions in cell survival during normal and pathological conditions. 

The expression of Prdx5 is closely related to the tumor size, depth, and lymphatic invasion in patients suffering from gastric cancer [177]. Moreover, the enhanced expression of Prdx5 leads to augmented carcinogenicity by increasing the proliferation and invasiveness of gastric cancer cells through the upregulation of Snail [177]. The treatment of Hodgkin’s lymphomas is based on targeting ROS, but the increased expression of mitochondrial Prdxs leads to chemoresistance [178]. Increased levels of Prdx5 have been observed in aggressive Hodgkin’s lymphomas [178]. In breast cancer, Prdx5 is upregulated in the mammary tissues and it is associated with poor prognosis. GATA1 transcription factor binds to the promoter region of Prdx5 in breast cancer cells and downregulates the transcription of Prdx5 [179]. The overexpression of Prdx5 protects cancerous cells from oxidative stress-induced apoptosis in a GATA1-regulated manner [165,179]. This implies that GATA1-regulated Prdx5 transcription can be targeted to treat breast cancer, but it requires further analysis involving the overexpression of GATA1 and its effects on tumor production and metastasis. Like Prdx3, the expression of Prdx5 is upregulated in endometrial cancer and this enhanced expression of Prdx5 in the endometrium of females with endometrial tumor can serve as a prognostic marker [167]. Mitochondrial Prdxs are overexpressed in ovarian cancer cells, and Prdx5 serves as a negative predictor of survival in patients suffering from ovarian cancer [180,181,182]. The upregulated expression of Prdx5 is also observed in malignant mesothelioma cells [169], while a reduction in Prdx5 expression has only been described in adrenocortical carcinoma [183]. 

In summary, the Prdx5 is upregulated in different cancers, except for adrenocortical carcinoma, but which factors are controlling the upregulation of Prdx5, the pathways associated with Prdx5 upregulation and cancer cell survival are lacking and need further investigation to demonstrate the exact mechanism of action of Prdx5 in different cancers and to develop strategies for effective drug designing.

## 8. Mitochondrial Prdxs and Chemoresistance

Cancerous cells are unique when compared with normal cells, in that they include elevated ROS levels as well as an increased level of antioxidants to counterbalance the ROS [111]. This distinctive feature of cancerous cells is attributable to the development of resistance in cancerous cells against chemo and radiotherapy, as these therapies are highly dependent on ROS-developed cytotoxicity [114]. A plethora of literature has described the association of elevated levels of Prdxs with chemo- or radioresistance to various drugs [184,185,186,187,188]. 

In breast cancer, the upregulated expression of Prdx3 is associated with the development of resistance to the drug doxorubicin [189]. Prdx3 regulates the apoptotic signaling pathway by controlling the release of cytochrome c from the mitochondria, along with establishing the linkage with leucine zipper kinase and IKB kinase [165]. Therefore, it will be a good strategy to develop drugs that target Prdx3 and mitochondrion specific electron suppliers, i.e., thioredoxin2 (Trx2), thioredoxin reductase2 (TrxR2), and sulfiredoxin (Srx), for response improvement of different chemotherapeutic agents, such as cisplatin, paclitaxel, and etoposide [189,190]. In many other cancers, such as breast cancer, ovarian cancer, and erythroleukemia, chemoresistance is developed by the upregulated expression of Prdx1, Prdx3, and Prdx6 [191]. In addition, there is evidence that Prdx5 is also involved in chemoresistance to adriamycin, bleomycin, vinblastine, and dacarbazine in patients of Hodgkin’s lymphoma and in vitro lung carcinoma U1810 cell lines [192]. 

**Figure 5:**
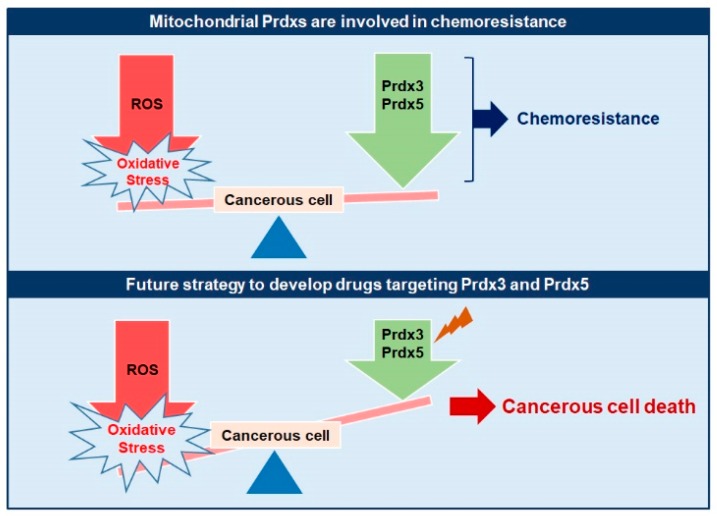
Mitochondrial Prdxs in development of Chemoresistance. The upregulated expression of mitochondrial Prdxs is associated with the development of drug resistance in a number of cancer types leading to complications in cancer treatment. These upregulated Prdxs can be specifically targeted in cancerous cells to develop new drugs against cancer with better efficacy and without causing any harm to the normal cells.

Chemoresistance is a complicated process that involves different factors and numerous modes of action affected by the tumor microenvironment as well as tumor biology [193,194]. Alterations in endogenous antioxidants play a determining role in the development of chemoresistance, as well as can serve as promising targets to design new drugs with better efficacy [195] (Figure 5). 

## 9. Concluding Remarks 

Mitochondrial Prdxs, the multifunctional proteins of the cells, are well known for their physiological as well as pathological significance based on their interplay with ROS. The crosstalk between ROS and mitochondrial Prdxs is critical for the initiation and progression of various types of cancers. The targeting of mitochondrial Prdxs for developing drugs against cancer is a good strategy. However, because these Prdxs are associated with chemoresistance in certain cancers, it is conceivable that, instead of targeting mitochondrial Prdxs, it will be better to diagnose the signaling pathways and microenvironments of these Prdxs in particular cancer types and then develop a drug that can target the root, leading to the activation of these Prdxs in response to the elevated ROS levels.

## 10. Future Directions

Our survey of studies analyzing the involvement of mitochondrial Prdxs in human cancers shows that most of the conducted studies are based on the expression analysis of Prdx3 and Prdx5. It is depicted that mitochondrial Prdxs are upregulated in variety of cancer types and directly or indirectly regulated by transcription factors, microRNAs, and oncogenes. Further, the interaction of Prdx5 with response elements is also reported, but current studies for analyzing the roles of mitochondrial Prdxs and ROS in cancer need more in depth analyses, as described below

1. Most of the studies reporting the upregulation of mitochondrial Prdxs in human cancers are cell-line specific, it will be more advantageous to design in-vivo studies to explore the interaction of these Prdxs in cellular environment.
2. Mitochondrial Prdxs have the ability to function as molecular chaperons, enzyme activators, and can be involved in protein-protein interactions beyond their enzymatic peroxidase functions. Accordingly, the transgenic animal (mouse) models should be used to demonstrate the role of mitochondrial Prdxs in oncogenic signaling. It will be beneficial to understand the exact mechanism of action of these Prdxs in cancer and to design effective drugs targeting a particular pathway associated with cancer survival and progression.
3. Although a plethora of studies describe the regulation of mitochondrial Prdxs by different transcription factors, oncogenes, and microRNAs in different types of cancer, but the exact mechanism of mitochondrial Prdxs in different types of cancers and their upstream and downstream regulators is lacking. Incorporation of variety of omics techniques i.e., transcriptomics, proteomics, and metabolomics into the in vitro and in vivo studies of mitochondrial Prdxs in cancer development can help to elucidate signaling mechanism in future studies.
4. More clinical investigations are needed to evaluate the differences in the expression of Prdx3 and Prdx5 between normal and diseased state. In addition, the expression of mitochondrial Prdxs during the early and late stage of cancer should be analyzed to demonstrate their role as anti-oncogenic or pro-oncogenic in different cellular context.
5. The upregulated expression of Prdx3 and Prdx5 is associated with the development of chemoresistance in different cancers and selective targeting of these mitochondrial Prdxs can lead to sensitization of cancer cells to chemotherapy. This fact should be investigated in detail to unveil the underlying mechanisms.

## Abbreviations

ROS	Reactive oxygen species
Prdxs	Peroxiredoxins
ATP	Adenosine triphosphate
SOD	Superoxide dismutase
Cys	Cysteine
Cp	Peroxidatic cysteine
Cr	Resolving cysteine
ERK	Extracellular signal related kinase
JNK	Jun N-terminal kinase
MAPK	Mitogen activated protein kinase
PTP	Protein tyrosine phosphatases
PTEN	Phosphatase and tensin homologues
CSCs	Cancerous stem cells
NRF2	Nuclear factor erythroid 2 related factor
KEAP1	Kelch like ECH associated proteins
AREs	Antioxidant responsive elements
HIFs	Hypoxia inducible factors
PHDs	Prolyl hydroxylases
pVHL	von Hippel-Laundau protein
CCRCC	Clear cell renal cell carcinoma
FOXM1	Forkhead box protein 1
LNCaP	Lymph node carcinoma of prostate
TIMP1	TIMP metallopeptidase inhibitor 1
ECM	Extracellular matrix
HepG2	Human hepatocellular carcinoma/hepatoma cell lines
MCF-7	Mitichigan cancer foundation-7 cancerous cell lines
HER2	Human epithelial growth factor receptor 2
Trx2	Thioredoxin 2
TrxR2	Thioredoxin reductase 2
Srx	Sulfiredoxin
MM	Malignant mesothelioma
NF-κB	Nuclear factor κB
ARE	Antioxidant responsive element
IRE	Insulin responsive element
GRE	Glucocorticoid responsive element
Ets ½	E twenty six transcription factor 1 and 2
HMGB1	High mobility group protein B1
